# Consistency and flexibility in solving spatial tasks: different horses show different cognitive styles

**DOI:** 10.1038/s41598-017-16729-z

**Published:** 2017-11-29

**Authors:** Paolo Baragli, Valentina Vitale, Claudio Sighieri, Antonio Lanata, Elisabetta Palagi, Adam R. Reddon

**Affiliations:** 1 Department of Veterinary Sciences, University of Pisa, Pisa, Italy; 2 Unitat Equina, Fundació Hospital Clìnic Veterinari, Universitat Autònoma de Barcelona, Barcelona, Spain; 3 Department of Information Engineering & Research Center “E. Piaggio”, School of Engineering, University of Pisa, Pisa, Italy; 4 Museum of Natural History, University of Pisa, Pisa, Italy; 5 Institute of Cognitive Sciences and Technologies, National Research Council, Rome, Italy; 6 Department of Biology, McGill University, Montréal, Canada; 7 Present Address: School of Natural Sciences and Psychology, Liverpool John Moores University, Liverpool, UK

## Abstract

Individual animals vary in their behaviour and reactions to novel situations. These differences may extend to differences in cognition among individuals. We tested twenty-six horses for their ability to detour around symmetric and asymmetric obstacles. All of the animals were able to get around the barrier to reach a food target, but varied in their approach. Some horses moved slowly but were more accurate in choosing the shortest way. Other horses acted quickly, consistently detoured in the same direction, and did not reliably choose the shortest way. The remaining horses shifted from a faster, directionally consistent response with the symmetric barrier, to a slower but more accurate response with the asymmetric barrier. The asymmetric barrier induced a reduction in heart rate variability, suggesting that this is a more demanding task. The different approaches used to solve the asymmetric task may reflect distinct cognitive styles in horses, which vary among individuals, and could be linked to different personality traits. Understanding equine behaviour and cognition can inform horse welfare and management.

## Introduction

Intraspecific variation in behaviour is ubiquitous among animals^1,2^. Individuals that are proactive^3^, bolder, more aggressive, and faster exploring^4^ can potentially gather rewards more rapidly^5^. Conversely, shy, unaggressive, slow exploring^4^ and reactive animals^3^ might be safer, but collect fewer rewards, at least in the short-term^5^. This variation in behaviour may affect the response to cognitive challenges at the individual level^6,7^. For example fast-exploring animals learn operant conditioning tasks more quickly (*Poecile atricapillus* ^8^, *Brachyrhaphis episcopi* ^9^), whereas slow-explorers perform better in reversal learning (*Poecile atricapillus* ^10^, *Parus major* ^11^) and avoidance learning tasks (*Parus major* ^12^). Therefore, the outcome of a single test cannot provide sufficient information on the adaptive value of the cognitive strategies employed^13^. For instance, animals that solve tasks quickly may also make more mistakes (i.e. a speed-accuracy trade-off^14^). Broadly speaking, fast animals act to maximize short-term gains, whereas slower animals take time to make more accurate decisions. Hence, neither speed alone nor accuracy alone is necessarily adaptive, and a range of different problem solving strategies may yield similar fitness payoffs^5^. Position on this speed accuracy trade-off (SAT) may help to define an animal’s individual cognitive style^15^, which may in turn, be related to personality at individual level^6^.

Orienting oneself in a dynamic environment is vital for animals to maintain access to resources, for example, when the direct route to a target is blocked and a new path must be found. These detour problems require animals to select alternative routes to reach a target and have been widely used to study spatial problem solving in animals^16,17^. The ability to find the best route to a reward implies that the animal is able to determine the distance, direction and timing required to reach the goal^16,18^.

Cognitive challenges, including spatial problems, may induce a physiological reaction as a consequence of emotional arousal^19^. These responses may be related to the behavioural characteristics of the individual^3^. Behavioural variation has frequently been associated with specific patterns of autonomic nervous system (ANS) activity^20^. When subjected to psycho-physiological challenges, shy animals tend to exhibit hesitancy or anxiety, while bold animals show excitement^3^. In both cases, the sympatho-vagal balance of the ANS is altered to cope with the situation^20^. Consequently the sympatho-vagal balance can be used as physiological marker of psychological states in animals^19,21^. The series of beat-to-beat time intervals is obtained by computing the temporal distance between consecutive R waves of the QRS complex of an electrocardiogram^22^. Heart Rate Variability (HRV) is related to the antagonistic influences of the sympathetic and parasympathetic branches of the ANS on the cardiac sinoatrial node^21^. Therefore the HRV is a simple, reliable, and non-invasive tool for investigating the sympatho-vagal balance, thus providing a quantitative measure of emotional arousal^23^.

The economic and social importance of horses has stimulated considerable scientific interest. Horses are proficient at using spatial information to locate food and other rewards^24,25^ and therefore, are a good species to test hypotheses about the link between behavioural variation and cognitive styles when animals have to cope with spatial challenges. In a previous paper designed to test for lateralization and detour ability in horses, we found that some individuals tend to behave more cautiously, while others made quick decisions at the expense of accuracy^24^. Based on this prior work, we hypothesize that horses use different strategies to solve spatial tasks reflecting different cognitive styles.

In the current study, we exposed adult female horses to each of two different detour tasks, one with a symmetric and one with an asymmetric barrier between the horses and a food reward. Cognitive styles should be evidenced by consistency in response across two tasks (symmetric *vs* asymmetric barriers). Based on a speed - accuracy trade off, the cognitive style used should affect the time required to solve the task. We also expect that horses that use different strategies would show distinct patterns of sympathetic/parasympathetic balance on heart control, reflected by differences in HRV.

## Methods

### Animals

We used 26 female Standardbred horses (age 10.8 ± 3.5 years) for this study. These animals were employed as receivers in the embryo transfer program where the study was performed (Department of Veterinary Sciences, University of Pisa, Italy). All subjects were trained and handled from the left side as is conventional for domestic horses and were housed in small groups in four paddocks (40 m × 20 m) with ad libitum access to hay and water (see Supplementary Fig. S1). Due to the peculiarity of the embryo-transfer program, our study horses were not exposed to a stable social context as defined by Krueger & Flauger^26^.

### Apparatus

**Figure 1:**
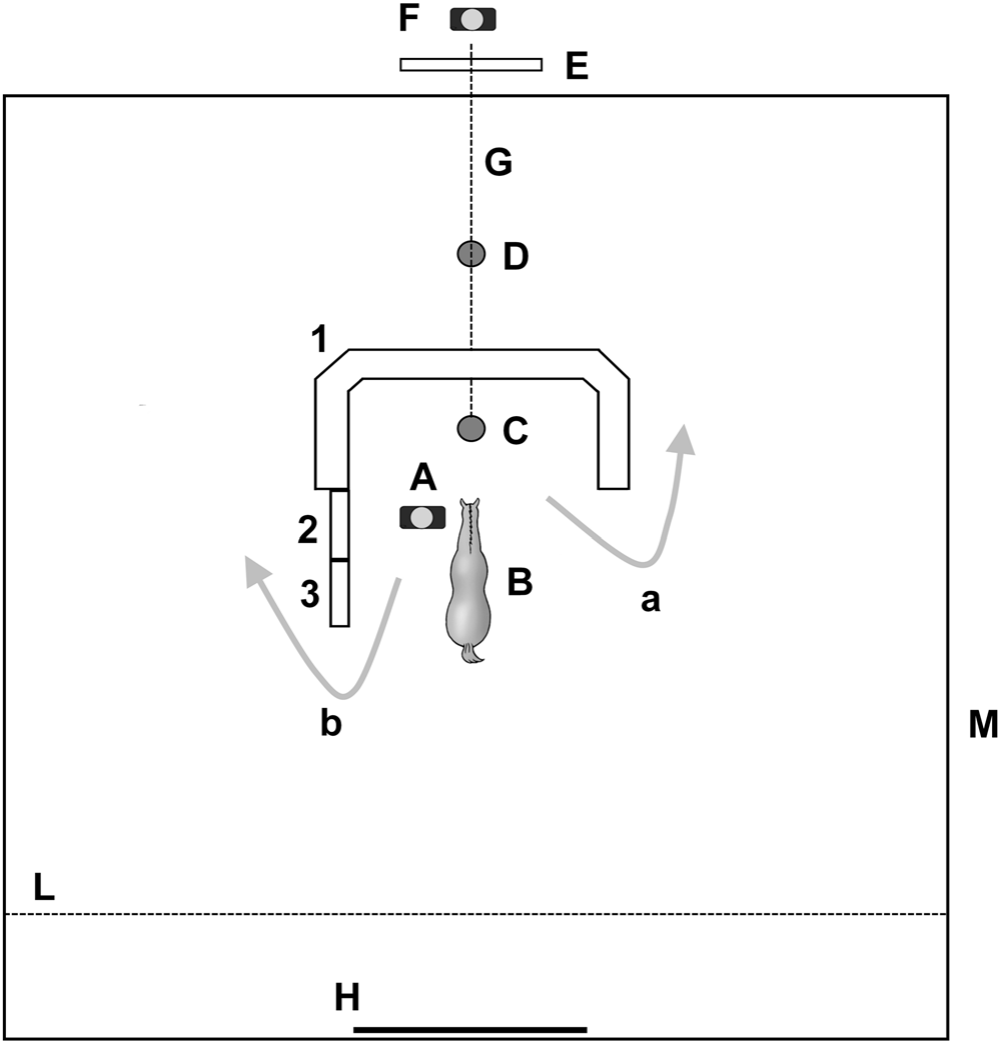
Apparatus used. A = experimenter who held the horse; B = starting position of the horse (position in which the horse was released); C = starting position of food bucket; D = final position of food bucket and horse after detouring obstacle; E = wooden panel outside the enclosure; F = experimenter who pulled the trolley and recorded the detour time; G = rope to pull the trolley; H = gate; L = invalidation line (positioned at 2 m from the entrance side of the enclosure); M = enclosure (16 × 16 m); 1 = U-shape obstacle; 2 and 3 = wooden panel (100 × 100 cm) added to base of obstacle to create asymmetry in Detour task 2. This figure has been already published on Baragli, P., Vitale, V., Paoletti, E., Sighieri, C., & Reddon, A. R. (2011). Detour behaviour in horses (*Equus caballus*). Journal of Ethology, 29(2),227–234 and is reproduced here with permission of Japan Ethological Society and Springer Japan.

We used two different barriers in a detour task. First, we constructed a symmetric, U-shaped barrier by stacking blocks of stable-litter wood shavings in the middle of a small enclosure (Fig. 1). The barrier allowed horses to see over from the opposite side and maintain visual contact with the food reward. An opening at ground level, located in the centre of the barrier, enabled the food reward to be transferred from the inner part of the barrier to the outside. The food reward was kept in a white bucket and placed on a square trolley that could be pulled by a cord through the barrier. To create an asymmetric barrier, we used two wooden panels (one meter each in length), which were added to one of the two arms of the barrier to create a long and a short way around^24^. The added barriers doubled the distance that the horses needed to cover when going the long way around the asymmetric barrier.

### Experimental design

After being familiarized with the apparatus to avoid a fearful reaction to the novel barrier, each horse was tested with each of the two barriers. The horses first performed 15 trials of the symmetric task, followed by 15 trials of the asymmetric task for a total of 30 trials per horse. Each horse underwent 5 consecutive trials per day for a period of 6 days. The sequence of tasks was held constant in order to determine if and how the asymmetrical barrier affected the detour strategy and heart rate variability of the study horses when compared with the symmetrical condition, which served as a baseline^27^.

At the beginning of each trial, an experimenter led the horse by a rope attached to the halter, towards the barrier. The position of experimenter (left/right side of the horse) leading the horse by the halter was alternated in each trial. The horse was released at the edge of the barrier while the experimenter turned and departed the way they came. The experimenter did not accompany the animal to the target food bucket. The horse was allowed to eat a small amount of the food before a second experimenter, hidden outside the enclosure, pulled the trolley and food bucket from the inner side of the barrier, to approximately 200 cm on the far side of the barrier. The second experimenter began pulling the bucket toward the opposite side of the barrier only after the first experimenter had left the enclosure. No one remained within sight of the horse during the trial. When the horse reached the target object beyond the barrier she was allowed to eat all of the food remaining in the bucket. If the horse crossed the invalidation line (Fig. 1), the task was repeated. The position of the added barriers during asymmetric task was determined by tossing a coin with the constraint that the added barriers could not remain on the same side for more than two consecutive trials^27^. The detour was considered successful if the horse reached the food bucket on the other side of the barrier within 5 min from the moment the bucket was pulled through the barrier.

For each successful trial, we recorded the latency to reward as the time in seconds from the moment the reward trolley passed through the breach to the moment the horse put its muzzle in the bucket after detouring the barrier. In the asymmetric detour task, we recorded whether the horse chose the shorter or the longer way around the barrier. To verify changes in the detour direction between symmetric and asymmetric barrier (to the right or left of the horse’s initial position) we computed the Laterality index (LI = [detours to the right − detours to the left]/[detours to the right + detours to the left]) for each horse, separately for each of the two detour tasks^28^. Horses that performed 15 detours on the same side of the barrier (LI =  ±1) in either of the detour tasks were considered to be directionally consistent, while horses that performed at least 1 detour on the other side of the barrier (−1 < LI < 1) within each of the detour tasks were considered to be directionally flexible.

Prior to the daily testing session, each horse was fitted with a modified apex-base ECG telemetry system monitor (Life Scope 8; Nihon Kohden, Tokyo, Japan) fixed by a chest belt, which remained on for the entire session of trials for that day. The ECG telemetry system was equipped with rubber electrodes thus avoiding the use of adhesive materials and shaving the skin. The ECG of horses was acquired with a sampling frequency of 1000 Hz by the NI/USB-6009 acquisition board equipped with Signal Express software (National Instruments, Austin, TX, USA). After a semiautomatic procedure for visual inspection and editing^22^, the ECG signal was then offline processed using proprietary software where the Stationary Wavelet Transform decomposition algorithm was applied to identify and remove movement artefacts from ECG traces in horses^29^.

This allowed us to obtain the inter-beats (RR, i.e. the distance between consecutive R waves of the QRS complex of the electrocardiogram) time intervals in milliseconds (ms). Its variability is known to be related to the antagonistic influences of the parasympathetic and sympathetic branches of the ANS control on the cardiac sinoatrial node. Across each 5-trial daily block, we selected the time interval of 5 minutes corresponding to the best stationary condition of the RR series^22^. Then a time domain analysis was performed and the standard deviation of the mean RR series (SDNN, ms), and root mean square of successive squared RR differences (RMSSD, ms) were computed with Kubios HRV software^30^. The SDNN and the RMSSD are the simplest parameters used to estimate overall and short-term variability related to parasympathetic activity^21^, thus reflecting the integrity of vagus nerve-mediated autonomic control of the heart. Specifically, RMSSD is associated with short-term, rapid changes in heart rate, and with the electrical stability of heart influenced by the parasympathetic nervous system’s activity^31^. Generally, a reduction of the SDNN parameter indicates a reduction in dynamic complexity of HRV, whilst lower RMSSD indicates a reduction of the parasympathetic control of cardiac activity. Both reductions refer to more regular HRV series, which usually is a marker of higher sympathetic activity, compared to parasympathetic activity^21,22^.

### Statistical analysis

We evaluated which factors could explain the variation in the latency to reward, SDNN and RMSSD via a generalized linear mixed-model (GLMM) analysis. Latency to reward, SDNN and RMSSD were the dependent variables. The fixed factors for each GLMM analysis are reported in Table 1. The latency to reward was distributed according to the Gamma function (Log-link), whereas SDNN and RMSSD were normally distributed (Shapiro-Wilk test for normality, ns; Anderson–Darling, ns; Easy Fit 5.5 Professional). To be conservative, we used robust model estimation to handle violations of model assumptions. We tested models for each combination involving the variables of interest (Table 1), spanning from a single-variable model to a model including all the fixed factors (full model). We also included all possible interactions between factors. To select the best model, we used the Akaike’s corrected information criterion (AICc), which adjusts Akaike’s information criterion (AIC) for small sample sizes. As the sample size increases, AICc converges to AIC. To measure the fit of the best model compared to the next best model, we calculated the difference (Δi or ΔAICci) between the AICc value of the best model and the AICc value for each of the alternative models^32^. Moreover, to assess the relative strength of each candidate model, we employed Δi to calculate the evidence ratio and the Akaike weight (wi). The evidence ratio provides a measure of how much more likely the best model is than the model i. The wi (ranging from 0 to 1) is the weight of evidence or probability that a given model is the best model, taking into account the data and set of candidate models^32^. We examined the fold change between the HRV measured in symmetric and asymmetric tasks (ΔSDNN and ΔRMSSD). We checked for possible correlations between i) the laterality index and the number of times the horse selected the shortest way (correct choice), ii) the number of correct choices and ΔSDNN, and iii) the number of correct choices and ΔRMSSD. Then we tested for possible correlations across trials using randomization procedures to avoid pseudo-replication due to non-independence of data. Specifically, randomization tests were performed using resampling procedures with 10 000 permutations. Due to the non-normal distribution (Shapiro-Wilk test for normality, p < 0.05) and the small sample size, non-parametric statistics were used^33^ to compare the difference in laterality index between symmetric and asymmetric tasks and choice of short and long way in the asymmetric task. The binomial test was used to analyse the choice of short and long way for each horse in the asymmetric task (analysis within each individual). The analyses were performed using SPSS 20.0.0 (SPSS, Chicago, IL, USA) and Resampling Procedures 1.3 package (by David C. Howell). We set the significance level to p < 0.05, while also discussing results with p values > 0.05 and <0.1 as potential trends.

**Table 1:** Description of the variables used in GLMM analyses.

NAME	TYPE
***Dependent Variable***	
Latency to reward	Scale
SDNN	Scale
RMSSD	Scale
***Fixed Explanatory Variables***	
Barrier type (symmetric/asymmetric)^(1,2,3)^	Dichotomous (0 = symmetric; 1 = asymmetric)
Trial order (ranging from 1 to 15)^(1)^	Ordinal
Laterality index (LI)^(1,2,3)^	Scale
Δ between LI_symmetric_ and LI_asymmetric_^(1,2,3)^	Scale
Day order^(2,3)^	Ordinal (ranging from day 1 to day 3)
Age in years^(1,2,3)^	Scale
***Random Variables***	
Horse ID^(1,2,3)^	Nominal

^(1)^Variables considered for the latency to reward; ^(2)^Variables considered for SDNN; ^(3)^Variables considered for RMSSD.

### Ethical notes

This study was carried out in accordance with the EU Directive 2010/63/EU for animal experiments (adopted by the Italian Animal Care Act, decree Law 26/2014). The Ethical Committee on Animal Experimentation of the University of Pisa approved the experimental protocol (ref. n. 63714).

## Results

In the symmetric detour task, we observed 12 directionally flexible horses, while the other 14 horses were directionally consistent. In the asymmetric detour task, 8 of the directionally consistent horses in the symmetric task shifted their approach and detoured at least once in each direction, thus becoming directionally flexible in the asymmetric task. The remaining 6 horses continued to be directionally consistent, regardless of the asymmetric barrier (4 detouring to the right and 2 detouring to the left). The whole study group showed a tendency (P = 0.055) to change their laterality index between the two tasks (see Supplementary Table S1 for individual laterality index).

**Table 2:** Best GLMM models for each target variable.

**Models for the dependent variable:** ***latency to reward***	**AICc**	**ΔAICc**	***W*** **i**
Barrier type; Trial order; Laterality index	1370.18	0	0.5157
Barrier type; Trial order; Laterality index; Δ between LI_symmetric_ and LI_asymmetric_	1370.93	0.748	0.3548
Barrier type; Trial order; Laterality index; Age	1374.10	3.916	0.0728
**Models for the dependent variable:** ***SDNN***	**AICc**	**ΔAICc**	***W*** **i**
Barrier type; Day order; Laterality index; Δ between LI_symmetric_ and LI_asymmetric_; Age	1395.97	0	0.8540
Barrier type; Day order; Laterality index; Δ between LI_symmetric_ and LI_asymmetric_	1399.66	3.694	0.1347
**Models for the dependent variable:** ***RMSSD***	**AICc**	**ΔAICc**	***W*** **i**
Barrier type; Day order; Laterality index; Δ between LI_symmetric_ and LI_asymmetric_; Age	1223.94	0	0.4938
Barrier type * Laterality index; Day order; Δ between LI_symmetric_ and LI_asymmetric_	1225.34	1.4	0.2452
Barrier type; Day order; Laterality index; Δ between LI_symmetric_ and LI_asymmetric_	1225.67	1.732	0.2077
Barrier type * Laterality index; Day order; Δ between LI_symmetric_ and LI_asymmetric_ * Age	1229.36	5.422	0.0328

To define the trade-off of GLMM the best model/s with ΔAICc below 2 and the first of model with ΔAICc above 2 have been included for each target variable (latency to reward, SDNN and RMSSD).

**Figure 2:**
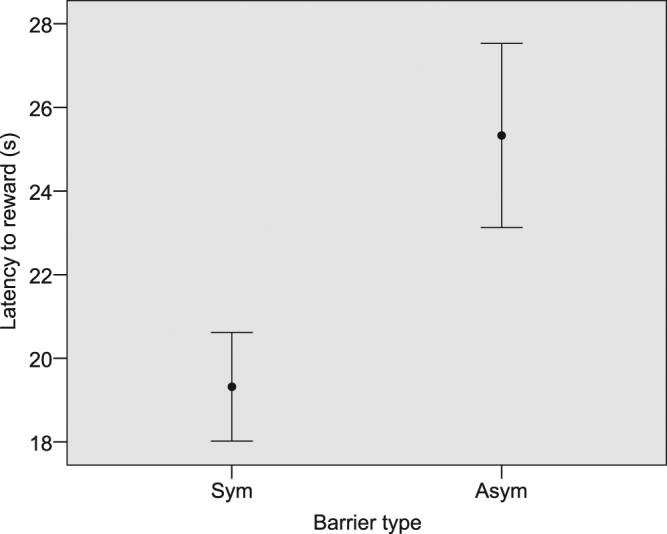
Difference in the latency to reward between symmetric and asymmetric conditions (p = 0.0001).

**Figure 3:**
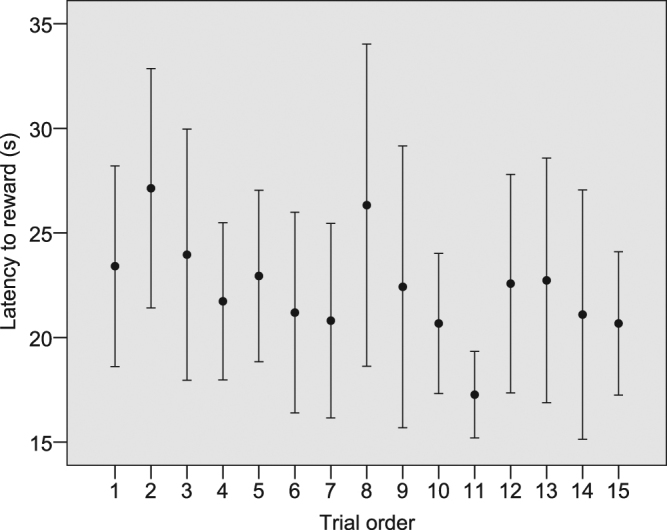
Effect of trial number on the latency to reward (p = 0.043).

**Figure 4:**
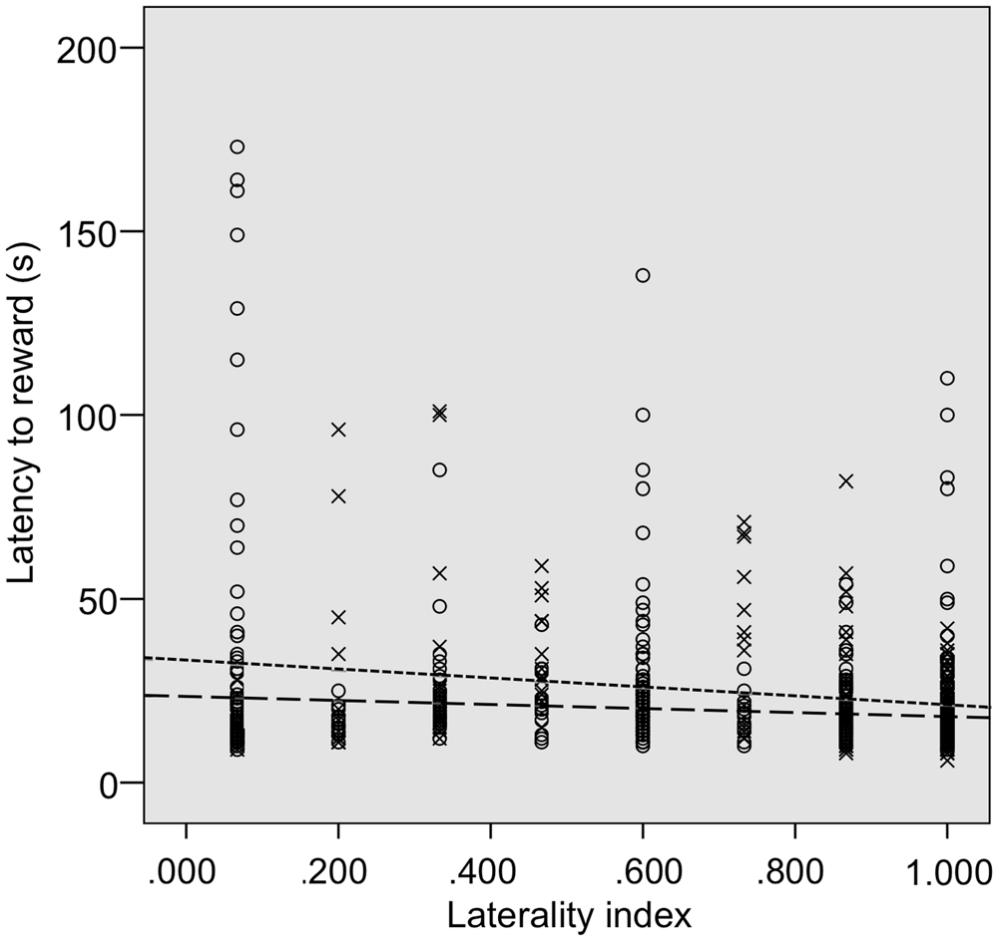
Negative correlation between laterality index and latency to reward in both symmetric (X marker, long dashed line; p = 0.006) and asymmetric conditions (O marker, short dashed line; p = 0.0001).

Regarding the time the horses spent to successfully navigate the detour and reach the reward on the opposite side (latency to reward), we found two competing models of the GLMM analysis that cannot be discarded due to their ΔAICc less than 2, while a third model had its ΔAICc above 2 (Table 2). Respectively, the first model explained 51.57%, the second one explained 35.48% of the variance in latency to reward, while the third model explained only 7.28%. In the two competing models, the variables barrier type and trial order were significant (Table 3). The latency to reward increased with asymmetry (Fig. 2), while for both types of barrier the latency to reward decreased as the trials proceeded (Fig. 3). In both models we found a statistical trend for laterality index (Table 3). In order to evaluate if this trend was reliable for both the symmetric and asymmetric tasks, we calculated the correlation via a randomization test. We found a negative correlation between the latency to reward and laterality index in both symmetric (r = −0.137; n_trials_ = 388; p = 0.006) and asymmetric tasks (r = −0.179; n_trials_ = 389; p = 0.0001; Fig. 4).

**Table 3:** Best GLMM models explaining the distribution of latency to reward, SDNN and RMSSD in horses.

*Latency to reward*				
Fixed variables (first model, AICc = 1370.18)	*F*	*df1*	*df2*	*P*
Barrier type	30.101	1	760	0.000*
Trial order	1.746	14	760	0.043*
Laterality index	3.443	1	760	0.064^#^
**Fixed** **variables (second model, AICc = 1370.93)**	***F***	***df1***	***df2***	***P***
Barrier type	30.133	1	759	0.000*
Trail order	1.746	14	759	0.043*
Laterality index	3.375	1	759	0.067^#^
Δ between LI_symmetric_ and LI_asymmetric_	0.001	1	759	0.976
***SDNN***				
**Fixed variables (first model, AICc = 1395.97)**	***F***	***df1***	***df2***	***P***
Barrier type	15.915	1	137	0.000*
Δ between LI_symmetric_ and LI_asymmetric_	2.413	1	137	0.123
Age	0.554	1	137	0.458
Day order	0.472	2	137	0.625
Laterality index	0.105	1	137	0.747
***RMSSD***				
**Fixed variables (first model, AICc = 1223.94)**	***F***	***df1***	***df2***	***P***
Day order	3.387	2	137	0.037*
Barrier type	2.613	1	137	0.108
Laterality index	1.585	1	137	0.210
Δ between LI_symmetric_ and LI_asymmetric_	1.406	1	137	0.238
Age	0.369	1	137	0.545
**Fixed variables (second model, AICc = 1225.34)**	***F***	***df1***	***df2***	***P***
Day order	3.405	2	138	0.036*
Barrier type * Laterality index	1.698	2	138	0.187
Δ between LI_symmetric_ and LI_asymmetric_	0.923	1	138	0.338
**Fixed variables (third model, AICc = 1225.67)**	***F***	***df1***	***df2***	***P***
Day order	3.356	2	138	0.038*
Barrier type	2.694	1	138	0.103
Laterality index	1.715	1	138	0.192
Δ between LI_symmetric_ and LI_asymmetric_	1.399	1	138	0.239

Significance of fixed variables within the best models (*P ≤ 0.050; ^#^P ≤ 0.100).

**Figure 5:**
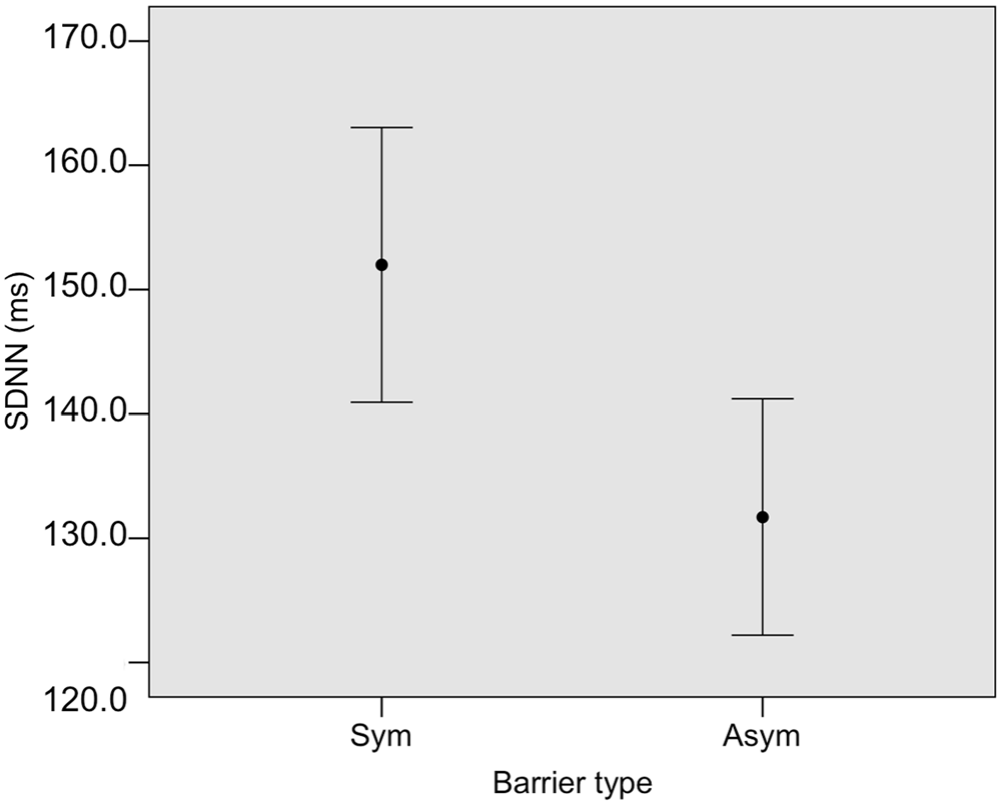
The SDNN parameter significantly decreased in asymmetric condition respect to the symmetric one (p = 0.0001).

Turning our attention to HRV, we ran a GLMM to examine which factors influenced the SDNN (Table 1). The best model explained 85.40% of the variance in SDNN, while the second model explained 13.47% (Table 2). Only the barrier type remained significant (Table 3); the SDNN was higher in the symmetric compared to the asymmetric task (Fig. 5).

**Figure 6:**
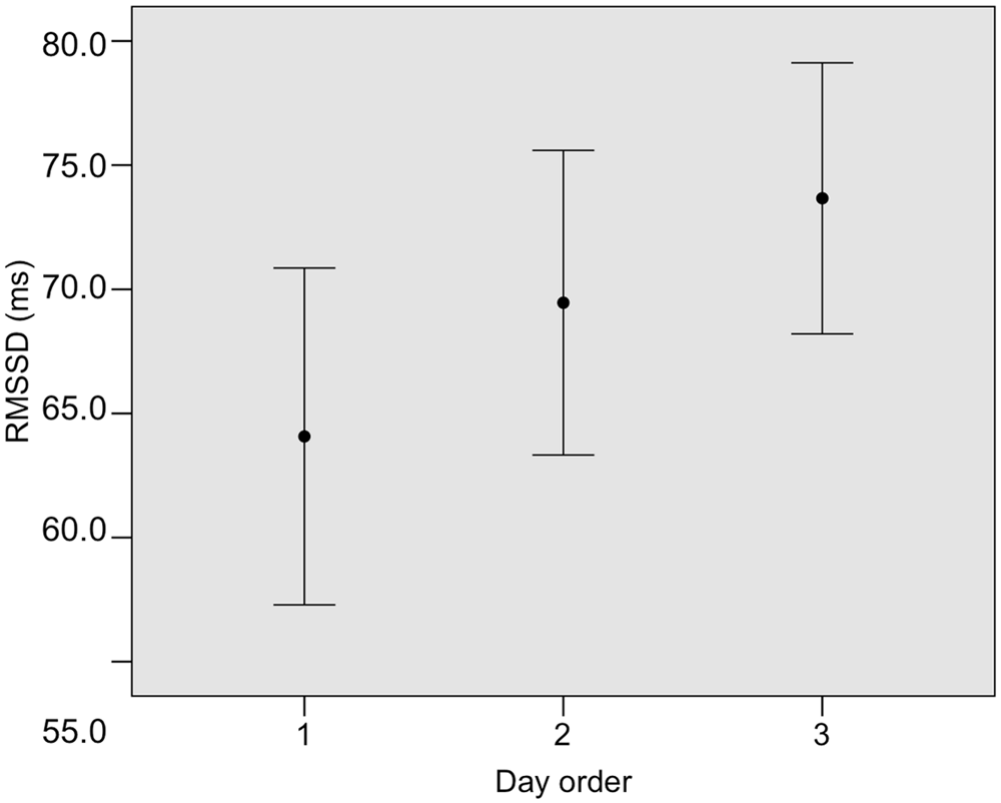
The RMSSD parameter increased with testing day (from day 1 to day 3 in both symmetric and asymmetric conditions).

To examine which factors influenced the RMSSD we also ran a GLMM (Table 1). We found three competing models that cannot be discarded due to their ΔAICc less than 2, while the fourth model had its ΔAICc above 2 (Table 2). Respectively, the first model explained 49.38%, the second explained 24.52% and the third explained 20.77% of the variance in RMSSD, while the fourth model explained 3.28% of the variance in RMSSD. Day order was significant in all the three best models (Table 3); the RMSSD increased from the first to the third day in both symmetric and asymmetric task (Fig. 6).

**Figure 7:**
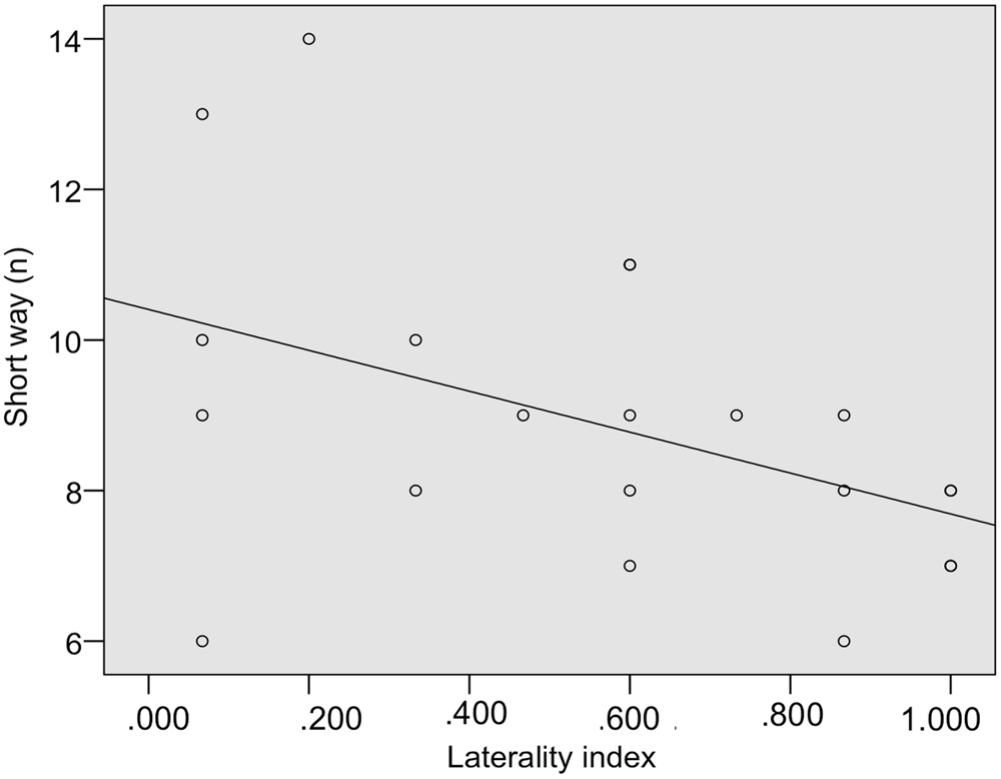
Laterality index and the choice of the shorter way are negatively related (p = 0.013) in the asymmetric condition.

With the symmetric barrier some horses were directionally flexible and they maintained this behavioural strategy with the asymmetric barrier. The remaining horses were directionally constant with the symmetric barrier. Some of them changed their behavioural strategy with the asymmetric barrier, thus becoming directionally flexible. Therefore horses that were directionally flexible with the asymmetric barrier included horses that maintained the same strategy as they enacted in the symmetric task and horses that changed their strategy as the barrier changed. These directionally flexible horses (that varied their detour direction) in the asymmetric task significantly chose the short way at group level with two horses that consistently chose the short way at individual level (see Table 4 for the choice of shorter way at individual and group level). We found a negative correlation between the laterality index and the number of times the horse selected the shorter way (Pearson r = −0.480, n_horses_ = 26, p = 0.013; Fig. 7). We did not find any correlation between the number of choices of the short way and either ΔSDNN (Pearson r = 0.132, n_horses_ = 26, p = 0.522) or ΔRMSSD (Pearson r = 0.252, n_horses_ = 26, p = 0.214). All relevant data are included (Supplementary Data).

## Discussion

In the present study, female Standardbred horses showed the ability to reach a food reward in spatial detour tasks with both symmetric and asymmetric barriers. The tested horses showed individual differences in their approach to solving these detour tasks, which may vary with their underlying personality. Horses improved at each detour task over successive trials, reaching the reward more quickly as the task progressed. The latency to reward was higher in the asymmetric than in the symmetric task, thus suggesting that the asymmetric barrier was more challenging. The changes in the sympatho-vagal balance on heart control revealed a reduction of vagal control in the asymmetric task, suggesting an increased arousal when coping with the more difficult task. The variability of the RMSSD increased across testing days in both symmetric and asymmetric tasks, suggesting decreased arousal as the horses gained more experience with the task.

Some horses were directionally flexible (−1 < LI < 1; Supplementary Table 1) in both the symmetric and asymmetric detour tasks, and were slower to reach the reward in both cases. In the asymmetric task, these directionally flexible horses tended to select the shorter way more often. It is possible that more time was necessary for these horses to assess the test environment^34^. In a spatial task, reactive, slow exploring animals typically sample multiple aspects of the environment and build an accurate map of the area^5^. These animals are usually more sensitive to environmental variation and behave in a shy manner^5^. These personality traits are generally linked to choices that require more time but are also more accurate^35^. The higher latency to reward together with the more frequent choice of the shorter route showed by the directionally flexible horses that varied their detour direction in the asymmetric task suggests a problem solving approach that favours accuracy over speed.

Regardless of the task, some horses detoured the barrier on the same side in each trial. These individuals reached the reward faster and may have used a heuristic approach, based on previous success, or behaved according to an inherent directional bias^25^. Consistency in detour direction has previously been reported for several animal species^27,28,36,37^. These directional predispositions, such as turning biases may be driven by cerebral lateralization, which is the preferential use of one hemisphere of the brain over the other to process sensory information, make a decision and generate motor outputs^38^. Cerebral lateralization is a widespread phenomenon among vertebrates and can influence cognition by affecting how individuals acquire, process, and store information^39,40^. Fixed behavioural outcomes such as consistent direction in detour tasks could be the consequence of prevalent action of one hemisphere. Meanwhile flexibility of the final action could be the average outcome of processing information with both hemispheres^41^. As with other mammals, the horses show lateral biases^42–44^, which are linked with the reaction to environmental stimuli^45,46^.

Several authors have argued that lateralized processing may be advantageous in cognitive or spatial tasks^47–49^. However, the strategy adopted by these horses is suboptimal in terms of the distance travelled to the reward and could be related to impulsiveness, which is the tendency to prefer immediate over delayed rewards^50^. Impulsiveness tends to be associated with boldness, favouring speed over accuracy, and with proactive tendencies (*Homo sapiens* ^50^, *Pan paniscus* and *Pan troglodytes* ^51^, *Rattus norvegicus* and *Columbia livia* ^52^). These personality traits are often linked with strongly lateralized behaviours and brains^41^. The directionally consistent horses in both tasks may ignore the changing environmental information, represented in our case, by the introduction of the asymmetric barrier. These individuals appear to perform routine sets of behaviour resulting in a tendency to stick to an option even if it is not optimal^53,54^.

Eight horses showed a directionally consistent approach in the symmetric task (LI =  ±1) but detoured the barrier on both sides in the asymmetric task, thus becoming directionally flexible. Therefore, at least some horses seemed to be able to modify their response to the changing task demands. These horses solved the symmetrical task more quickly than those that detoured in both directions and tended to choose the shorter way more often in the asymmetric task (Table 4). These eight horses, may show the optimal balance of speed and accuracy, using a quick heuristic approach (directionally consistent detouring) when the choice is irrelevant, but switching to a more considered approach when one of the options entails an additional cost. The cognitive plasticity^55^ shown by these horses allowed them to adjust their response thus reducing the costs (time and distance) to reach the reward. These horses demonstrated the ability to detect the change in the environment, to inhibit the action plan followed in the symmetric task and to select a more appropriate action in the asymmetric task. The different behavioural responses shown by these horses in the two tasks suggest that the speed-accuracy trade-off alone cannot explain all aspects of cognitive style^7^ in this species.

**Table 4:** Choice of the shorter way performed by each horse in the asymmetric condition.

Horse ID	Short Way (out of 15)	Long Way (out of 15)	Binomial Test	Wilcoxon’s signed ranks test^a^
H8 (1)	14	1	z = 3.1 p = 0.001*	
H17 (1)	13	2	z = 2.58 p = 0.0074*	
H1 (1)	11	4	z = 1.55 p = 0.1185	
H5 (1)	10	5	z = 1.03 p = 0.3018	
H24 (1)	10	5	z = 1.03 p = 0.3018	
H3 (1)	9	6	z = 0.52 p = 0.6072	
H22 (1)	9	6	z = 0.52 p = 0.6072	
H19 (1)	8	7	z = 0 p = 1.0	
H9 (1)	8	7	z = 0 p = 1.0	
H7 (1)	8	7	z = 0 p = 1.0	
H16 (1)	7	8	z = 0 p = 1.0	
H21 (1)	6	9	z = −0.52 p = 0.6072	
Mean ± SD	9.4 ± 2.4	5.6 ± 2.4		z = −2.407; p = 0.003
H26 (2)	11	4	z = 1.55 p = 0.1185	
H12 (2)	9	6	z = 0.52 p = 0.6072	
H14 (2)	9	6	z = 0.52 p = 0.6072	
H2 (2)	9	6	z = 0.52 p = 0.6072	
H11 (2)	8	7	z = 0 p = 1.0	
H10 (2)	8	7	z = 0 p = 1.0	
H20 (2)	7	8	z = 0 p = 1.0	
H6 (2)	6	9	z = −0.52 p = 0.6072	
Mean ± SD	8.4 ± 1.5	6.6 ± 1.5		z = −1.496; p = 0.047
H25 (3)	8	7	z = 0 p = 1.0	
H13 (3)	8	7	z = 0 p = 1.0	
H15 (3)	8	7	z = 0 p = 1.0	
H18 (3)	8	7	z = 0 p = 1.0	
H4 (3)	7	8	z = 0 p = 1.0	
H23 (3)	7	8	z = 0 p = 1.0	
Mean ± SD	7.7 ± 0.5	7.3 ± 0.5		z = −0.816; p = 0.688

Some horses (1) maintained the same cognitive style as in symmetric task; they were directionally flexible and detoured the barrier on both sides. Other horses (2) shifted from directionally consistent response in the symmetric task to directionally flexible response in the asymmetric task. The remaining horses (3) maintained the same cognitive style and were directionally consistent in both tasks. Therefore in the asymmetric task horses (1) and (2) were directionally flexible showing the same cognitive style. These directionally flexible horses (1) and (2) significantly selected the shorter way; with two horses from the (1) who significantly selected the shorter way at individual level*. ^a^Asymp. Sig. (2-tailed).

Regarding the physiological data, the SDNN in the asymmetric compared to the symmetric task indicated a reduction of vagal tone (increased sympathetic activity) on heart control, suggesting that the asymmetric task elicited greater emotional arousal^19,54,56^. The horses may experience an anxiety-like mood when faced with the novel asymmetric barrier. The RMSSD data suggest increased vagal control of heart activity over successive testing days within each task. This observation suggests habituation to the task in both the symmetric and the asymmetric conditions. Furthermore, the reduction of the latency to reward across trials may also suggest habituation to the task with increasing familiarity or practice. The RMSSD and the latency to reward data suggest that the reduced SDNN in the asymmetric task may not be anxiety-related, but rather represents emotional arousal due to excitement about the reward^57,58^. The reduction of vagal activity in the asymmetric task could also be affected by physical exertion^20^. When horses chose the longest way, they may walk faster to reach the reward as quickly as possible. These explanations are not mutually exclusive, and the increased sympathetic activity recorded in asymmetric task may have multiple interrelated causes. It is worth noting that SDNN and RMSSD do not provide information on the valence of the emotional response experienced (positive or negative), and therefore alternative interpretations are possible.

We found that a change in the environment (the introduction of the asymmetric barrier) has consequences for the detour strategy, the latency to reach the reward, and on the activation of autonomic nervous system. Collectively, these factors may define a cognitive style^5,54^ that varies among individual horses and affects their efficiency in gathering resources. We speculate that these cognitive phenotypes likely influence, and are influenced by, the personality of the horse, though future studies will be needed to test this hypothesis. Establishing the cognitive style and personality of a horse would be beneficial for equine management and training. Studies on horse temperament^59^ are typically performed through human judgment^60^, reaction tests^61^ or both^62^. However, reports based on subjective appraisals are vulnerable to implicit bias, and repeatability issues are prevalent in the reaction tests that are routinely performed^63^. Therefore, improved behavioural and cognitive testing methods for horses would be an asset for horse management strategies. Our approach may therefore have practical applications for the welfare of domestic horses.

## Conclusion

Some of our horses were directionally flexible and they solved the detour tasks slowly, detoured in both directions in both tasks and were more accurate in choosing the shortest way in the asymmetric task. This could indicate shier, more reactive animals. By contrast, other horses moved quickly, were directionally consistent, and did not reliably choose the shortest way around the asymmetric barrier. Such horses may be bolder, more proactive, and/or impulsive. Finally, some horses seem to occupy an intermediate position between slower/more accurate and faster/less accurate responses. These horses shifted from a faster, directionally consistent response in the symmetric task to a slower more accurate and directionally flexible approach in the asymmetric task. These individuals may exhibit the optimal strategy in such spatial tasks and this sort of cognitive plasticity could correlate with overall success in navigating spatial challenges, which may translate into fitness benefits. Understanding the cognitive style and its variation together with personality of individual horses has important implications for the training and management of these animals. By integrating cognitive measures with personality and physiology, our study offers a novel window onto the inter-individual differences in equine behaviour and cognition.

## Electronic supplementary material

Supplementary Figure 1

Supplementary Table S1

Supplementary Dataset
