# Supplementary material for: Consistency and flexibility in solving spatial tasks: different horses show different cognitive styles
Source: Sci Rep. 2017 Nov 29;7:16557. doi: 10.1038/s41598-017-16729-z (PMC5707407; doi:10.1038/s41598-017-16729-z)
Supplement: Supplementary file 1 — Supplementary Figure 1 [file 41598_2017_16729_MOESM1_ESM.pdf]

Consistency and flexibility in solving spatial tasks: different horses show different cognitive styles

Paolo Baragli [1,4 *], Valentina Vitale [2], Claudio Sighieri [1], Antonio Lanatà [3], Elisabetta Palagi [4,5], Adam R Reddon [6,7]

1 Department of Veterinary Sciences, University of Pisa, Italy.

2 Unitat Equina, Fundació Hospital Clìnic Veterinari, Universitat Autònoma de Barcelona, Spain.

3 Department of Information Engineering & Research Center "E. Piaggio", School of Engineering, University of Pisa, Pisa, Italy.

4 Museum of Natural History, University of Pisa, Italy.

5 Institute of Cognitive Sciences and Technologies, National Research Council, Rome, Italy.

6 Department of Biology, McGill University, Montréal, Canada.

7 Current address: School of Natural Sciences and Psychology, Liverpool John Moores University, Liverpool, UK.

Corresponding author:

*Paolo Baragli

Department of Veterinary Sciences, University of Pisa, Viale delle Piagge, 2, 56124, Pisa (Italy)

Email: paolo.baragli@unipi.it

Supplementary Figure 1

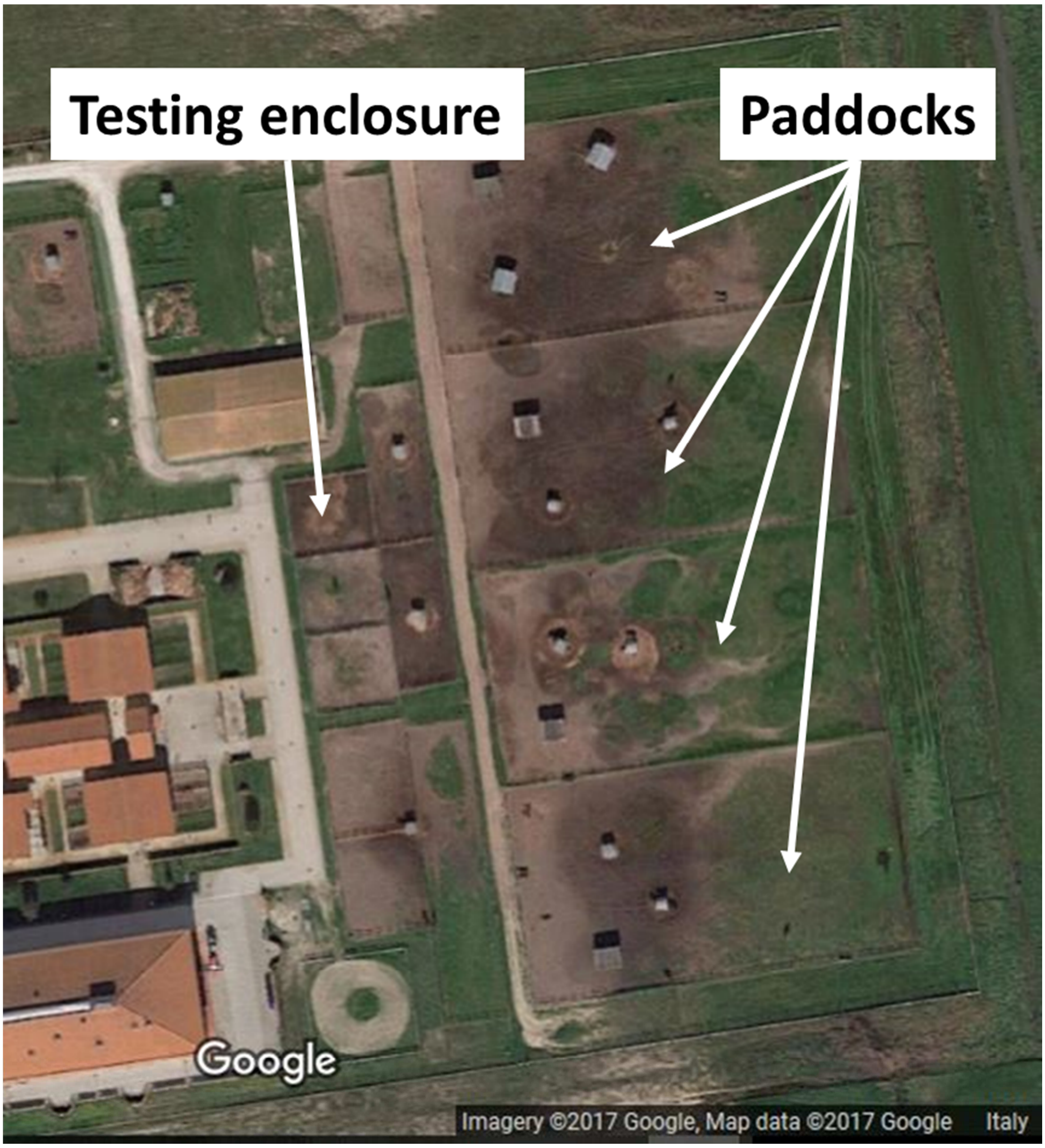

Aerial view of the equine facilities of the “Mario Modenato” Veterinary Teaching Hospital (Department of Veterinary Sciences, University of Pisa, Italy). The testing enclosure and paddocks where horses were housed are indicated (Map data ©2017 Google).
