# Supplementary material for: Consistency and flexibility in solving spatial tasks: different horses show different cognitive styles
Source: Sci Rep. 2017 Nov 29;7:16557. doi: 10.1038/s41598-017-16729-z (PMC5707407; doi:10.1038/s41598-017-16729-z)
Supplement: Supplementary file 2 — Supplementary Table S1 [file 41598_2017_16729_MOESM2_ESM.pdf]

Consistency and flexibility in solving spatial tasks: different horses show different cognitive styles

Paolo Baragli [1,4 *], Valentina Vitale [2], Claudio Sighieri [1], Antonio Lanatà [3], Elisabetta Palagi [4,5], Adam R Reddon [6,7]

1 Department of Veterinary Sciences, University of Pisa, Italy.

2 Unitat Equina, Fundació Hospital Clìnic Veterinari, Universitat Autònoma de Barcelona, Spain.

3 Department of Information Engineering & Research Center "E. Piaggio", School of Engineering, University of Pisa, Pisa, Italy.

4 Museum of Natural History, University of Pisa, Italy.

5 Institute of Cognitive Sciences and Technologies, National Research Council, Rome, Italy.

6 Department of Biology, McGill University, Montréal, Canada.

7 Current address: School of Natural Sciences and Psychology, Liverpool John Moores University, Liverpool, UK.

Corresponding author:

*Paolo Baragli

Department of Veterinary Sciences, University of Pisa, Viale delle Piagge, 2, 56124, Pisa (Italy)

Email: paolo.baragli@unipi.it

Table 1 Supplementary materials. Laterality index of each horse and relative changes between symmetric and asymmetric barrier.

Horse ID	Laterality Index (Symmetric barrier)	Laterality Index (Asymmetric barrier)	Wilcoxon's signed ranks test [a]
**H1 (1)**	0,867	0,600	
**H3 (1)**	0,467	0,067	
**H5 (1)**	0,867	0,067	
**H7 (1)**	0,733	0,867	
**H8 (1)**	0,333	0,200	
**H9 (1)**	0,867	1,000	
**H16 (1)**	0,200	0,600	
**H17 (1)**	0,333	0,067	
**H19 (1)**	0,067	0,333	
**H21 (1)**	0,067	0,867	
**H22 (1)**	0,867	0,467	
**H24 (1)**	0,067	0,333	
**Mean ± SD**	0.48 ± 0.34	0.46 ± 0.33	Z = -0.315 P = 0.753
**H2 (2)**	1,000	0,867	
**H6 (2)**	1,000	0,067	
**H10 (2)**	1,000	0,600	
**H11 (2)**	1,000	0,867	
**H12 (2)**	1,000	0,733	
**H14 (2)**	1,000	0,600	
**H20 (2)**	1,000	0,600	
**H26 (2)**	1,000	0,600	
**Mean ± SD**	1.00 ± 0.00	0.62 ± 0.25	Z = -2.555 P = 0.011
**H4 (3)**	1,000	1,000	
**H13 (3)**	1,000	1,000	
**H15 (3)**	1,000	1,000	
**H18 (3)**	1,000	1,000	
**H23 (3)**	1,000	1,000	
**H25 (3)**	1,000	1,000	
**Mean ± SD**	1.00 ± 0.00	1.00 ± 0.00	Z = 0 P = 1.000

(1) Directionally flexible horses who detoured the barrier on both sides; (2) horses shifted from directionally consistent bias to directionally flexible response; (3) horses which were directionally consistent in both task. [a] Asymp. Sig. (2-tailed) with normalized Laterality Index.
